# The predictive factors that are associated with the number of sutures used during meniscal repair

**DOI:** 10.1186/s12891-020-03911-0

**Published:** 2021-01-12

**Authors:** Xiaoxiao Song, Dongyang Chen, Xinsheng Qi, Qing Jiang, Caiwei Xia

**Affiliations:** 1 Department of Orthopedics, Affiliated Taikang Xianlin Drum Tower Hospital, Medical School of Nanjing University, Nanjing, Jiangsu People’s Republic of China; 2 Department of Sports Medicine and Adult Reconstructive Surgery, Nanjing Drum Tower Hospital, School of Medicine, Nanjing University, 321 Zhongshan Road, Nanjing, 210008 Jiangsu People’s Republic of China

**Keywords:** Meniscal repair, Sutures, Concurrent ACL injury, Lateral, Duration of injury

## Abstract

**Purpose:**

To investigate factors associated with the consumption of a large number of sutures during arthroscopic meniscus repair procedures.

**Methods:**

All patients who received meniscal repair, with or without concomitant anterior cruciate ligament (ACL) reconstruction, in our hospital from January 2015 to December 2019 were included in the current study. Demographic data (sex, age, body mass index (BMI), and injury-to-surgery interval) and surgical data (the site of the tear, side of the meniscus, presence of an ACL rupture or not and the number of sutures) were retrospectively collected from our medical records. The number of sutures was divided into two groups (1–2 sutures versus > 2 sutures). The stitching process was implemented through an all-inside technique using a meniscal repair device (Fast-Fix; Smith & Nephew). According to the length and stability of the meniscal tear, one to seven sutures were used. Univariate analysis consisted of chi-square tests. Multivariate logistic regression was then performed to adjust for confounding factors.

**Results:**

A total of 242 patients, including 168 males and 57 females, was finally included. In the univariate analysis, we found that those patients who underwent meniscus repair within one month after meniscus tear were more likely to need fewer sutures than those who underwent surgery more than one month after injury (70/110 versus 59/115, *p*=0.062). In total, 75/109 (68.8%) lateral meniscal tears were repaired with fewer sutures than medial (34/72, 47.2%) and bilateral meniscus injuries (20/44, 45.4%; *p*=0.003). In the multivariate analysis, we found that the duration of injury (OR, 2.06; 95% CI, 1.16–3.64, *p*=0.013), presence of an ACL injury (OR, 3.76; 95% CI, 1.97–7.21, *p*< 0.001) and the side of the meniscus (OR, 0.31; 95% CI, 0.14–0.65, *p*=0.002) were associated with the number of sutures used during meniscal repair procedures.

**Conclusions:**

Patients who underwent meniscal repair within one month after meniscus tear, especially lateral menisci tears, were more likely to need fewer sutures.

**Study design:**

Case-control study; level of evidence, 3.

## Background

The meniscus is the second stabilizer in the knee joint and can slow the progression of osteoarthritis (OA) by absorbing shock and transmitting load [1–3]. When meniscal tearing occurs, there are three options: nonoperative treatment, partial or total meniscectomy and meniscal repair. Nonoperative treatment has been regarded as an important choice to relieve knee pain and improve function, especially in patients with degenerative tears [4]. However, when the meniscal injury is serious such as bucket-handle tears or nonoperative treatment fails, an arthroscopic procedure must be performed. Many observational studies have demonstrated that meniscectomy can dramatically improve pain and knee function at short-term follow-up, but the loss of meniscal tissue also leads to the onset of early osteoarthritis [5, 6]. Compared with meniscectomy, meniscal repair can preserve meniscal tissue, thus restoring its biomechanical function and reducing the risk of developing knee OA in the future.

In the above setting, a consensus has been reached that meniscal tissue should be preserved as much as possible [1, 7, 8]. The decision as to whether to repair the injured meniscus is made by the surgeon. The indications for repair that surgeons usually consider include (1) the location of the meniscal tear and whether vascularity is adequate enough to enhance the rate of healing of the meniscus, and (2) the severity of the meniscal injury and whether the remaining meniscal tissue is adequate to make the repair viable [9].

The number of sutures is usually affected by the length of the meniscal tear. Theoretically, the longer the tear, the more sutures will be used [10, 11]. Patients often consult about the fee for the meniscal repair procedure preoperatively, but the surgeons cannot give them an accurate answer as it depends on the number of sutures, which are relatively expensive (560 dollars for one suture). Recently, in a study performed by John et al. [12], a greater number of sutures had been showed to be associated with a lower failure rate. Besides, the number of sutures indirectly reflect the length of meniscal tears which have been demonstrated to be a predicting factor of high failure rate of meniscal repair [2, 13]. Thus, we performed this study to investigate factors that are associated with a larger number of sutures so that we can inform patients about the probable surgical cost before surgery. Additionally, identifying these factors can also help surgeons to make surgical planning and counselling about prognosis preoperatively.

## Method

Institutional review board approval was waived. In the current study, 242 patients received meniscal repair, with or without concomitant anterior cruciate ligament (ACL) reconstruction, in our hospital from January 2015 to December 2019. All operations were performed by three highly experienced surgeons using a uniform procedure.

Demographic data (sex, age, body mass index (BMI), and injury-to-surgery interval) and intra-articular-related data (the site of the tear, the side of the meniscus, presence of an ACL rupture or not and the number of sutures) were retrospectively collected from our medical records. The injury-to-surgery interval was divided into an early group (≤1 month) and a delayed group (> 1 month). Age was classified into an older group (> 40 years) and a younger group (≤40 years), and BMI was classified into three groups (≤24 kg/m^2^, 24–27 kg/m^2^, and ≥27 kg/m^2^). The number of sutures was classified into two groups (1–2 sutures versus > 2 sutures).

All cases of meniscal tears were diagnosed via physical examination and magnetic resonance imaging (MRI) and confirmed by arthroscopic findings. Standard anterolateral and anteromedial knee portals were used for all patients undergoing meniscal repair under general anesthesia. After completion of diagnostic arthroscopy, a few steps were taken to repair meniscus. First, the meniscal disorder was defined, then both edges of the tear were trimmed and refreshed. Final, a meniscal repair device (Fast-Fix; Smith & Nephew) was used to finish the stitching process through an all-inside technique. According to the length and stability of the meniscal tear, one to seven sutures were used. If patients had a concomitant ACL rupture, ACL reconstruction would be performed using tendon of peroneus longus through an independent femoral approach fixed with a bioabsorbable interference screw as previously described [14].

An angle adjustable brace was used for all patients until 6 weeks after surgery. The angle of knee flexion was less than 45 degrees within 2 weeks after meniscal repair, 90 degrees within 4 weeks, and 120 degrees 6 weeks later and reach a similar angle as the uninjured side 8 weeks later after operation. Partial weightbearing was allowed until 4 weeks after surgery with the help of a crutch, and total weightbearing was encouraged 6 weeks after the operation.

### Statistical analysis

Statistical analyses were performed with SPSS, version 23.0 (SPSS Inc., Chicago, IL, USA). In the univariate analysis, we used the chi-squared test to describe the associations between categorical variables and the number of sutures used during meniscal repair. Risk factors with a *p* value less than 0.1 in the univariate analysis were used in the multivariate analysis. In the multivariate analysis, we used binary regression to determine the independent risk factors for a larger number of sutures. Odds ratios and 95% confidence intervals (CIs) were reported. A *p* value less than 0.05 indicated statistical significance.

## Results

In the current study, 242 patients, including 168 males and 57 females, with a mean age of 28.3 years (range from 10 to 63 years) who underwent meniscal repair in our hospital from January 2015 to December 2019 were included. Of these patients, 188 underwent concomitant ACL reconstruction, and 37 underwent isolated meniscal repair. The detailed data are shown in Table 1.

**Table 1:** Demographic baseline data

	≤2 sutures	> 2 sutures	*P*-value
Gender			0.603
Male	98	70	
Female	31	26	
Age			1.000
≤40	112	83	
> 40	17	13	
Weight			0.637
≤60	20	15	
60–90	96	65	
≥90	13	13	
Site of tear			0.620
Multiple	11	8	
Anterior	2	1	
Body	8	2	
Posterior	77	57	
ACL injury			0.311
Yes	105	83	
No	24	13	
Duration of injury			0.062
≤1 month	70	40	
> 1 month	59	56	
Side of meniscis			0.003
Lateral	75	34	
Medial	34	38	
Both	20	24	
Surgeons			0.311
1	58	45	
2	41	23	
3	30	28	

In the univariate analysis, we found that those patients who underwent meniscus repair within one month after meniscus tear were more likely to need fewer sutures than those who underwent surgery more than one month after injury (70/110 versus 59/115, *p*=0.062). In total, 75/109 (68.8%) lateral meniscal tears were repaired with fewer sutures than medial (34/72, 47.2%) and bilateral tears (20/44, 45.4%; *p*=0.003). In the multivariate analysis, we found that the duration of injury (OR, 2.06; 95% CI, 1.16–3.64, *p*=0.013), presence of an ACL injury (OR, 3.76; 95% CI, 1.97–7.21, *p*< 0.001) and the side of the meniscus (OR, 0.31; 95% CI, 0.14–0.65, *p*=0.002) were associated with the number of sutures used during the meniscal repair procedure (Table 2).

**Table 2:** Multivariable analysis of factors associated with the number of sutures

	Regression coefficient	95%CI	*P*-value
Duration of injury	2.06	1.16 to 3.64	0.013
Side of meniscis	0.31	0.14 to 0.65	0.002

## Discussion

Many articles have demonstrated that the loss of meniscal tissue can lead to early onset of degenerative knee joint changes in the long term. Verdonk et al. [15] and Hall et al. [16] claimed that the removal of 30% of meniscal tissue may increase joint surface contact forces by approximately 300%. Thus, a consensus has been reached that meniscal tissue should be preserved as much as possible [1, 7, 8]. The already popularized method to preserve meniscal tissue is the arthroscopic meniscus repair procedure.

With the development of meniscal repair surgery, many suture devices and techniques have been used [17, 18]. Several studies have been performed to investigate the clinical outcomes with different suture materials and repair techniques and have shown that the results are comparable with regard to the patient-reported outcomes and meniscal healing rate [19–21]. In our study, we used the FasT-Fix suture device for all patients with all-inside meniscal repair, which is the most widely used procedure. However, the FasT-Fix device is expensive. One of the most common questions that patients ask before surgery is how much they need to pay for the operation. However, that cannot be answered accurately, as the surgical cost is associated with the number of sutures. Furthermore, the number of sutures indirectly reflect the length of meniscal tears which have been showed to be a predicting factor of clinical prognosis [2, 13]. In some studies, authors demonstrated that length of meniscal tear less than 2 cm is a predicting factor for a lower failure rate [13]. John et al. [12] performed a study to investigate the risk factors associated with high failure rate of meniscal repair and found that patients with a greater number of sutures (2.97 sutures) are more likely to have a lower failure rate comparing those with a small number of sutures (1.79 sutures). Given the important clinical significance of the number of sutures, we implemented this study.

In the current study, we found that patients who underwent meniscal repair within one month after meniscus tear were more likely to use few sutures. The meniscus is an important second stabilizer of the knee joint. When it tears, especially in those patients with ACL deficiency, the knee is unstable [22, 23]. The meniscus becomes susceptible to additional force, particularly when surgery is delayed, and the incidence of subsequent meniscal and chondral lesions is significantly increased, which has been reported in a large number of studies [22, 24, 25]. Consequently, a serious meniscal tears, such as bucket-handle tears or long meniscal posterior root tears tend to consume a larger number of sutures. Additionally, our findings provide further evidence for the opinion that meniscal repair should be performed early.

Theoretically, medial menisci are more vulnerable to shear forces than lateral menisci. Because the medial meniscus is attached to the medial collateral ligament, its mobility is much smaller than that of the lateral meniscus, which may subsequently increase the severity of meniscal tears [26, 27]. Additionally, for most patients, arthroscopic surgery was delayed, which has been confirmed to be a potential risk factor for increased meniscal injury, especially in those patients with concomitant ACL rupture. Lateral meniscus tears are not associated with the injury-to-surgery interval [24]. The above two reasons may explain why the medial meniscus is more likely to need more sutures to be repaired.

Our results demonstrated that lateral meniscus injury and performing operations within one month after injury tend to require fewer suture devices. To our knowledge, this is the first study to investigate the risk factors that can increase the use of suture devices. We hope that identifying factors that are associated with the number of sutures used during meniscal repair can help surgeons to make surgical planning preoperatively. Besides, on the promise of having a good clinical outcomes, avoiding these risk factors, such as performing meniscal repair surgery within one month after meniscus injury, may have an influence on clinical prognosis.

However, our study has some limitations. First, this study was conducted based on data from a single medical center in China. We are not sure whether our findings can be generalized to the general orthopedic population in other hospitals. Nevertheless, we hope this study can provoke attention and thinking about what factors are associated with the use of a large number of sutures. Finally, all arthroscopic meniscal repairs were performed by three different highly experienced surgeons; thus, option bias may exist. However, we believe this study reflects realistic clinical issues.

## Conclusion

Those patients who underwent meniscal repair within one month after meniscus tear, especially lateral menisci tears, were more likely to need fewer suture devices.

## Data Availability

The datasets used and/or analyzed during the current study will be available from author XXS on a reasonable request and no material or illustrations have been previously published.

## Abbreviations

ACL: Anterior cruciate ligament
BMI: Body mass index
MRI: Magnetic resonance imaging
